# Increased Tim-3 expression on TILs during treatment with the Anchored GM-CSF vaccine and anti-PD-1 antibodies is inversely correlated with response in prostate cancer

**DOI:** 10.7150/jca.29705

**Published:** 2020-01-01

**Authors:** Xinji Zhang, Haixiong Chen, Guanfeng Li, Xiangyun Zhou, Yuqiang Shi, Feng Zou, Yuanxiang Chen, Jimin Gao, Shaomin Yang, Shihao Wu, Zhaolin Long

**Affiliations:** 1 Department of Urology, Shunde Hospital, Southern Medical University (The First People's Hospital of Shunde District, Foshan), Foshan, 528300, China;; 2 Department of Radiology, Shunde Hospital, Southern Medical University (The First People's Hospital of Shunde District, Foshan), Foshan, 528300, China;; 3 Zhejiang Provincial Key Laboratory of Medical Genetics, School of Life Sciences, Wenzhou Medical University, Wenzhou, 325035, China.

**Keywords:** programmed death receptor-1, T cell immunoglobulin and mucin domain-containing protein-3, prostate cancer, vaccine, immunotherapy.

## Abstract

Programmed death receptor-1 (PD-1) and T cell immunoglobulin and mucin domain-containing protein-3 (Tim-3) play important roles in tumor immune evasion. PD-1 blockade could produce an effective antitumor effect in many solid tumors except prostate cancer (PCa) because of rare programmed death ligand-1 (PD-L1) expression on PCa cells. Streptavidin (SA)-GM-CSF surface-anchored tumor cell (Anchored GM-CSF) vaccines could increase the number of tumor-infiltrating lymphocytes (TILs) and induce specific antitumor immune responses. The Anchored-GM-CSF vaccine and anti-PD-1 antibodies exerted synergistic effects in mouse models of PCa metastasis. However, the response rate was low due to the presence of other negative regulatory pathways. Tim-3 expression could be upregulated at resistance to combination therapy with anti-PD-1 antibodies and the Anchored GM-CSF vaccine. Sequential administration of anti-PD-1 and anti-Tim-3 antibodies could further improve the efficacy of the Anchored GM-CSF vaccine therapy, and tumor regression was noted in over 60% of animals. This triple therapy improved the specific cytotoxic activity, proliferation and secretion of CD8^+^ TILs and reduced the production of tumor-promoting cytokines. These findings indicated that this triple therapy could induce a robust antitumor immune response in mouse models of PCa.

## Introduction

Prostate cancer (PCa) is the most common cancer in males and the second leading cause of cancer death 1,2. Until now, available therapies for advanced stages of this disease are still limited, and their effectiveness is far from satisfactory.

In recent years, the field of cancer immunotherapy has seen remarkable growth, with the most notable development in T cell checkpoint inhibitors 3. Blockade of some of the regulatory molecules (especially programmed death receptor-1 (PD-1)/programmed death ligand-1 (PD-L1)) has been shown to be markedly effective in treating multiple cancer types except PCa 4. The reason is rare PD-L1 expression in primary PCa 5. PD-L1 is an IFNγ-responsive gene, and high PD-L1 expression indicates the existence of high levels of tumor antigen-specific IFNγ-secreting T cells 6. Therefore, pre-existing T cells specific for one or more tumor epitopes are used to determine the response to PD-1 blockade, further suggesting that checkpoint blockade might be more effective when combined with a method to increase the frequency of these tumor antigen-specific T cells.

In our previous studies, we developed a protein anchor platform to immobilize streptavidin (SA)-tagged bioactive molecules on the surface of biotinylated PCa cells and confirmed that the SA-GM-CSF-modified PCa cell (Anchored GM-CSF) vaccine could effectively induce a specific antitumor immunity in the RM-1 model 7. Furthermore, our recent study showed that the Anchored GM-CSF vaccine and anti-PD-1 antibodies exerted synergistic effects during PCa treatment 8. However, in this recent study, we found that tumor regression occurred in only a few mice and that the regression rate was low. This result was consistent with a recent clinical study, which found that targeting the PD-1 pathway did not always result in the reversal of T cell exhaustion 9. Several studies have demonstrated that PD-1 blockade could upregulate the expression of T cell immunoglobulin and mucin domain protein-3 (Tim-3) in head and neck cancer 10 and lung cancer 11. In addition, the level of upregulated Tim-3 expression was negatively correlated with the function of CD8^+^ T cells 12. The role of Tim-3 in the immune regulation of tumors, including PCa, has been confirmed by many studies 13-16.

Based on our previous studies and negative immunomodulation of Tim-3, in this study, we investigated Tim-3 expression during response or resistance to combined therapy with anti-PD-1 antibodies and the Anchored GM-CSF vaccine. Subsequently, we evaluated the efficacy of sequential administration of anti-PD-1 and anti-Tim-3 antibodies combined with the Anchored GM-CSF vaccine in long-established PCa mouse models.

## Methods

### Animals and cells

C57BL/6 mice (6- to 8-week-old) were purchased from the Experimental Animal Center of Southern Medical University (Guangzhou, China). All animal studies were performed in accordance with the National Institutes of Health guidelines for experimental animals (Ethical approval number: L2016045). The RM-1 cell line is a carcinogen-induced transitional cell carcinoma line derived from male C57BL/6 mice. RM-1 cells were cultured in RPMI 1640 medium containing 10% fetal bovine serum and 1% penicillin/streptomycin in a 5% CO_2_ humidified incubator. SA-GM-CSF and SA-green fluorescent protein (SA-GFP) fusion proteins were prepared at our laboratory.

### Vaccine preparation and bioactivity assay

According to our previous study 8, RM-1 cells were fixed in 30% ethanol (volume/volume) for 30 minutes and then incubated with 10 mM EZ-Link® Sulfo-NHS-LC-Biotin (Pierce Biotechnology, Rockford, USA) for 1 hour at room temperature. Then, the biotinylated RM-1 cells were incubated with SA-GM-CSF at 100 ng/10^6^ cells for 1 hour and washed 3 times with PBS. The percentage of SA-GM-CSF expression on the surface of RM-1 cells was assayed by flow cytometry using PE conjugated anti-GM-CSF monoclonal antibody.

The bioactivity of SA-GM-CSF immobilized on the surface of RM-1 cells was assessed by bone marrow cell proliferation. SA-mGM-CSF surface modified MB49 cells were pelleted and lysed by 3 freeze-thaw cycles. Membrane fractions were harvested by centrifugation and suspended in complete medium. Bone marrow and MTT were added for incubation. The reaction was stopped by adding dimethyl sulfoxide to each well. Absorbance was read at 570 nm on a microplate reader. SA-GFP was used as a negative control.

### Tumor model establishment and treatment

**Figure 1:**
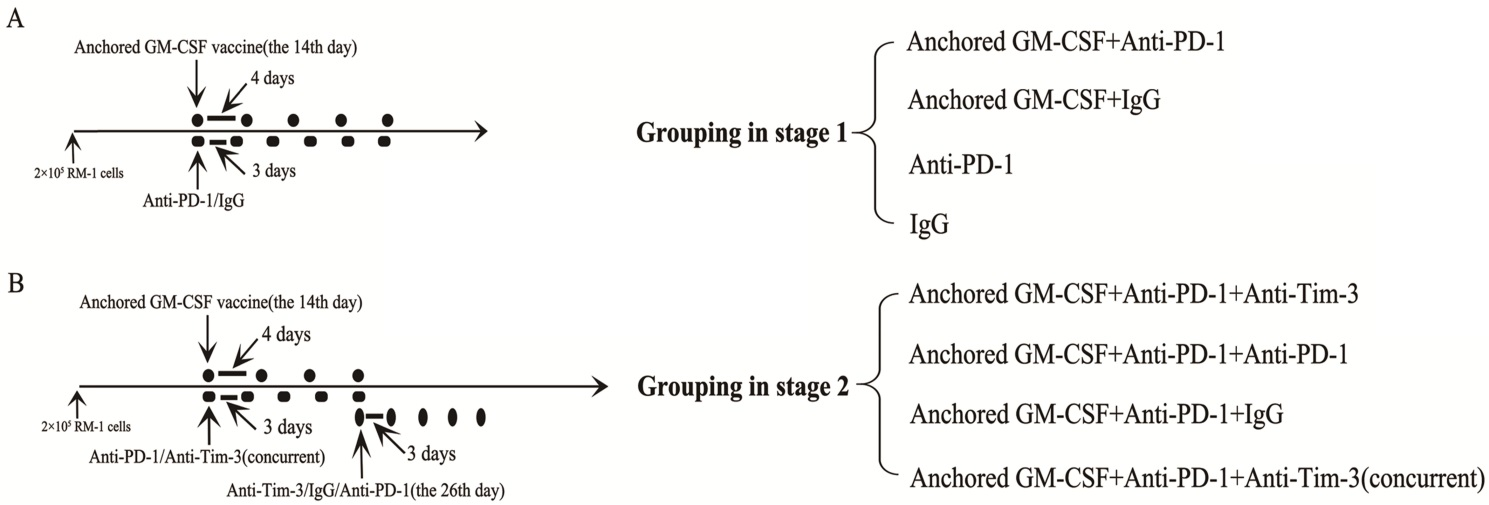
** Information on grouping and treatment.** (A) Grouping at stage 1, and detailed diagram of tumor therapy. (B) Grouping at stage 2, and detailed diagram of tumor therapy. The dosage and frequency of treatment: Anchored GM-CSF vaccine: 1×10^6^ cells/100 µl for each dose, once every 4 days. Anti-PD-1 antibody (eBioscience): 100 µg for each dose, once every 3 days. IgG (eBioscience): 100 µg for each dose, once every 3 days. Anti-Tim-3 antibody (Invitrogen): 100 µg for each dose, once every 3 days. The Anchored GM-CSF vaccine was administered by intracutaneous injection; the anti-PD-1 antibody, IgG, and anti-Tim-3 antibody were administered by intraperitoneal injection.

C57BL/6 mice were injected with 2×10^5^ RM-1 cells in the hind leg to establish a subcutaneous model. In contrast to our previous study, long-established subcutaneous models (2 weeks after RM-1 cells were injected) were used in the current study. This part of the study was divided into two stages. The first stage was to evaluate the efficacy of PD-1 blockade during treatment with the Anchored GM-CSF vaccine. The second stage was to evaluate the efficacy of sequential administration of anti-PD-1 (J43, eBioscience, USA) and anti-Tim-3 (RMT3-23, Bio X cell, USA) antibodies during treatment with the Anchored GM-CSF vaccine. Detailed information regarding the treatments is shown in Figure 1. Subcutaneous tumor growth was measured every three days, and the volume was calculated by the formula [π/6(w1×w2×w2)], where w1 and w2 represent the largest and smallest tumor diameters, respectively. The mice were sacrificed when the tumors were larger than 20 mm in their greatest dimension.

### Cytotoxicity assay

Splenocytes were isolated from each group of mice and stimulated by RM-1 cells as effector cells in the presence of interleukin (IL)-2 for 5 days. RM-1 cells seeded in 96-well plates served as target cells. Different numbers of effector cells were added to the target cells at the desired effector to target ratios. The supernatant was collected after 4 hours of incubation. Lactate dehydrogenase (LDH) activity was measured by the CytoTox 96® nonradioactive cytotoxicity assay (BD, USA). The experiment was repeated with MB49 bladder cancer cells (for specificity analysis).

### Immunohistochemistry (IHC)

Subcutaneous tumor tissues from each group of mice were collected and fixed with 4% paraformaldehyde and then embedded in paraffin. Paraffin sections (4-5 μm) were deparaffinized, rehydrated and treated with hydrogen peroxide, followed by antigen retrieval. After blocking for 10 minutes, the sections were incubated with anti-mCD4 (EPR19514, Abcam, UK) and anti-mCD8 (4SM16, eBioscience, USA) antibodies according to the instructions of the Rabbit-specific HRP/DAB (ABC, USA) Detection IHC Kit (Abcam, UK) and restained with hematoxylin and eosin.

### CD4^+^ and CD8^+^ T cell isolation

Fresh tumors or spleens from each group were cut into small pieces, transferred to 70-μm cell strainers (BD, USA) and separated using the plunger of a 5-ml syringe. The cells passed through the cell strainer were collected and subjected to Ficoll-Hypaque gradient centrifugation. After centrifugation, T cells were recovered and purified using CD4 or CD8 Microbeads (Miltenyi Biotec, CA).

### Flow cytometry

To evaluate CD4^+^ and CD8^+^ T cells in the peripheral environment (PE), we isolated splenocytes from each group and lysed red blood cells by FCM Lysing Solution (Multisciences Biotech, China). The splenocytes were washed with PBS and then stained with FITC-labeled anti-mCD4 (11-0042, eBioscience, USA) and APC-labeled anti-mCD8 antibodies (17-0081, eBioscience, USA). For the analysis of Tim-3 expression, we stained CD4^+^ and CD8^+^ T cells isolated from the tumor microenvironment (TME) and spleen with PE-labeled anti-Tim-3 antibody (B8.2C12, BioLegend, USA). For the analysis of proliferation, we collected CD8^+^ T cells and stained them with PE-labeled antibodies against Ki67 (11F6, BioLegend, USA) after permeabilization. For the analysis of secretion, we co-cultured CD8^+^ T cell subsets with anti-CD3-biotin (17A2, BioLegend, USA) and anti-CD28-biotin (37.51, BioLegend, USA). After permeabilization, these CD8^+^ T cells were stained with PE-labeled antibodies against IFNγ (XMG1.2, BioLegend, USA). Then, the above cells were analyzed by flow cytometry (Becton Dickinson, USA).

### Enzyme-linked immunosorbent assay (ELISA)

Peripheral blood (100 μl) was collected from each group and allowed to coagulate at room temperature for 20 minutes. The supernatants were harvested by centrifugation at 3000 rpm for 5 minutes. The concentrations of IL-6 and progranulin (PGRN) were measured by ELISA (Abcam, UK).

### Statistical analysis

All experimental group values were analyzed from representative experiments. The differences in tumor volume between groups were compared using repeated measures designs. Statistical analyses of flow cytometry, ELISA, and IHC data were performed by one-way ANOVA. Statistical analysis was performed using SPSS (version 19.0). *P*<0.05 was considered to be indicative of statistical significance.

## Results

### PD-1 blockade during treatment with the Anchored GM-CSF vaccine effectively reduced tumor growth in long-established models

**Figure 2:**
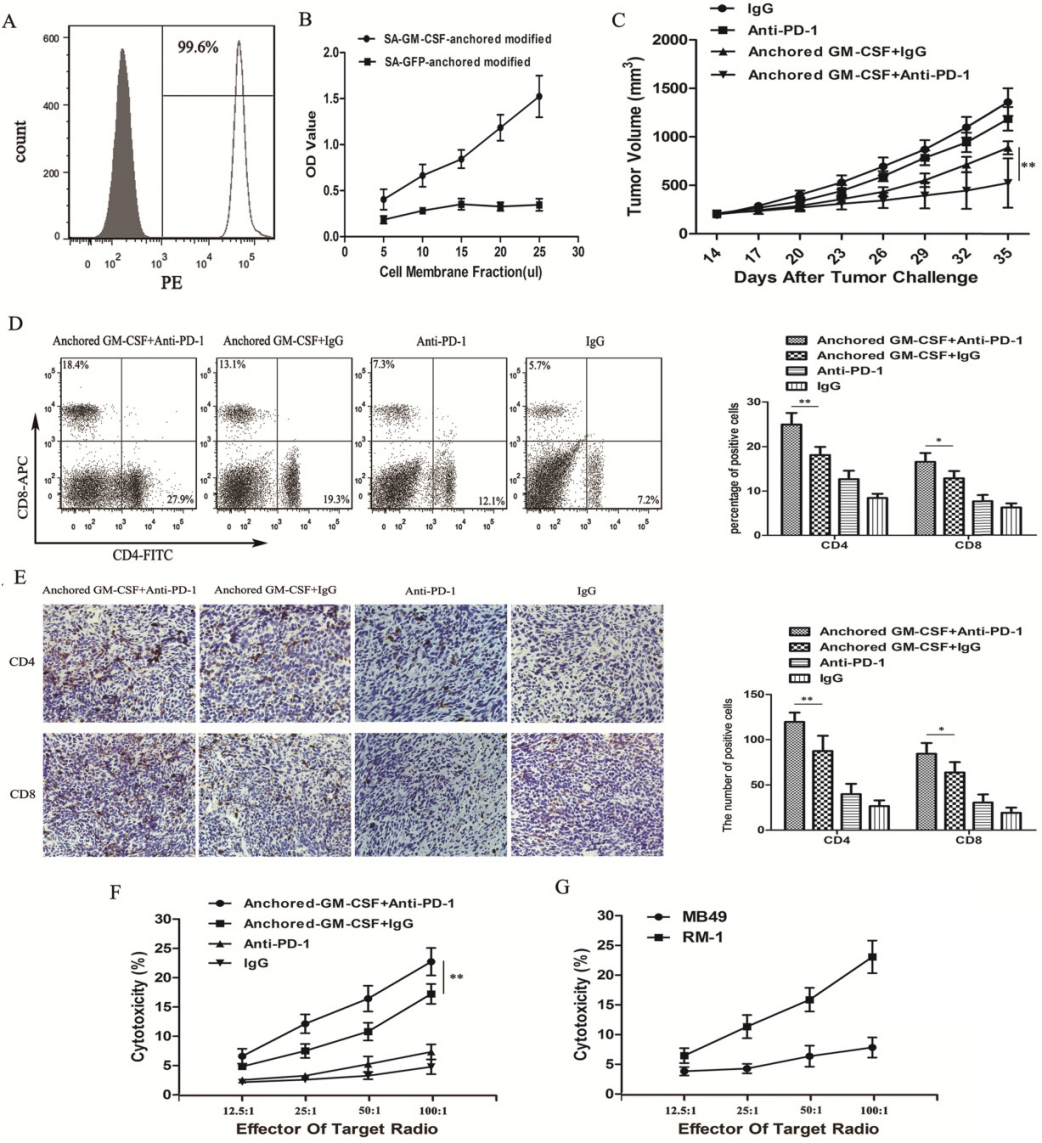
** Combination therapy with the Anchored GM-CSF vaccine and anti-PD-1 antibodies reduced tumor growth in long-established RM-1 models.** (A) The presence of SA-GM-CSF on the biotinylated surface of RM-1 cells was assayed by flow cytometry. Unbiotinylated RM-1 cells served as negative controls. (B) The proliferative effect of membrane-bound GM-CSF on bone marrow cells was assessed. OD, optical density. (C) All mice were subcutaneously injected with 2×10^5^ RM-1 cells, and the tumor volume was recorded. The combination therapy with the Anchored GM-CSF vaccine and anti-PD-1 antibodies effectively reduced tumor growth compared to the control treatment, but tumor regression occurred in only a few mice (3/8). In addition, there was no significant difference between the PD-1 blockade group and the IgG group in tumor growth inhibition (*P*>0.05). (D) The proportions of CD4^+^ and CD8^+^ T cell subsets in blood were measured by flow cytometry. (E) The proportions of CD4^+^ and CD8^+^ T cell subsets in TILs were measured by IHC. (F) Splenocytes were isolated from each group after treatment and stimulated with hIL-2. RM-1 cells served as target cells. Supernatants were collected for nonradioactive cytotoxicity assays. (G) To evaluate the specific cytotoxicity, splenocytes from the experimental group (Anchored GM-CSF vaccine+anti-PD-1 antibody) were isolated after treatment. RM-1 or MB49 cells served as target cells, and supernatants were collected for nonradioactive cytotoxicity assays. All experiments were repeated 3 times. ^*^P<0.05, ^**^P<0.01.

Flow cytometric analysis showed that SA-GM-CSF was efficiently anchored on the surface of RM-1 cells (Figure 2A) and retained bioactivity (Figure 2B).

To investigate the antitumor effect of the combination therapy with anti-PD-1 antibodies and the Anchored GM-CSF vaccine, we used long-established RM-1 models in this study (Figure 1A). Compared to the Anchored GM-CSF vaccine or anti-PD-1 antibody monotherapy, the combination therapy effectively reduced tumor growth and even promoted the regression of established tumors (Figure 2C). Notably, anti-PD-1 antibody treatment alone had no obvious therapeutic advantage over IgG (*P*>0.05).

Flow cytometry and IHC were used to detect the number of CD4^+^ and CD8^+^ T cells in the PE and TME. The two T cell subsets were obviously increased in the anti-PD-1 antibody and Anchored GM-CSF vaccine combination therapy group compared to those in the other groups (Figure 2D, E).

To further investigate antitumor immunity, we determined the extent of cytotoxicity in each group using LDH activity in each well. MB49 bladder cancer cells were used to further confirm specific antitumor immunity. Indeed, the combination therapy effectively improved the cytotoxic activity of cytotoxic T lymphocytes (CTLs) and promoted the establishment of tumor-specific T cell immunity (Figure 2F, G).

### Tim-3 expression was upregulated during resistance to combination therapy with anti-PD-1 antibodies and the Anchored GM-CSF vaccine

Although combination therapy with anti-PD-1 antibodies and the Anchored GM-CSF vaccine could effectively reduce tumor growth and improve the cytotoxic activity of CTLs relative to monotherapy, tumor regression occurred in only a few mice, and the majority of subcutaneous tumors continued to grow slowly. To further investigate the reasons for this phenomenon, we divided mice in the combined treatment group (Anchored GM-CSF vaccine+anti-PD-1 antibody) into sensitive and resistant subgroups depending on response to treatment. Resistance was defined as an initial therapeutic response of tumors followed by growth to >120% of the original tumor size.

**Figure 3:**
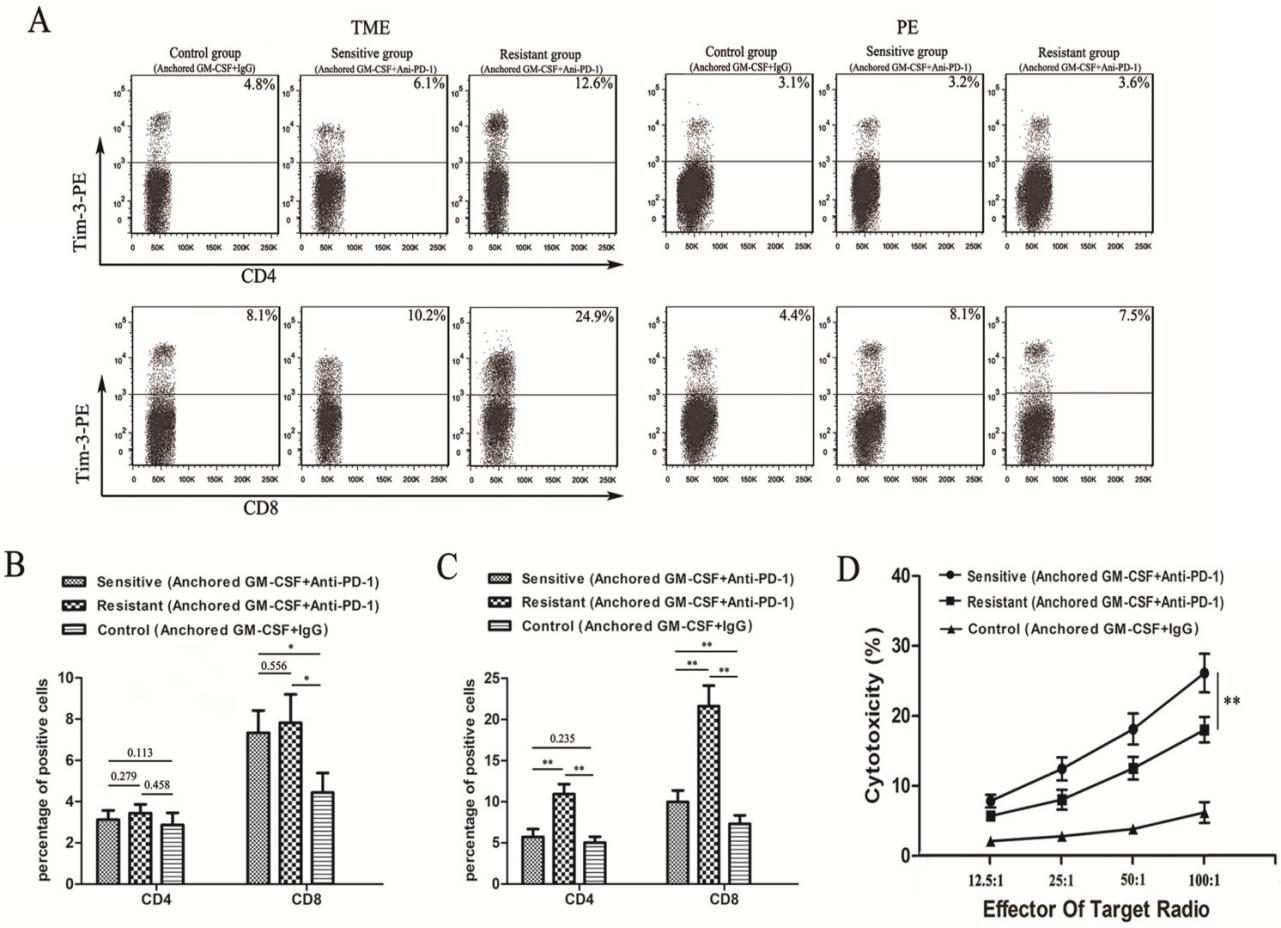
** Tim-3 expression and tumor-specific cytotoxicity assays in sensitive/resistant groups.** (A) After treatment, CD4^+^ and CD8^+^ T cells were isolated from tumor tissues and splenocytes from each group and purified by CD4 and CD8 Microbeads. Then, the T cell subsets were stained with antibodies against Tim-3, and Tim-3 expression was assessed by flow cytometry. (B, C) The proportions of Tim-3 expression on CD4^+^ and CD8^+^ T cells in the PE (B) and TILs (C) are shown in histograms. (D) Splenocytes were isolated from each group after treatment and stimulated with hIL-2. RM-1 cells served as target cells. Supernatants were collected for nonradioactive cytotoxicity assays. All experiments were repeated 3 times. ^*^P<0.05, ^**^P<0.01.

To confirm the expression of Tim-3 in different groups, we performed flow cytometry and found that Tim-3 was expressed at higher levels in both CD4^+^ and CD8^+^ T cells in the TME from the resistant group than in cells from the sensitive and control groups (Figure 3A). No difference in Tim-3 expression in either CD4^+^ or CD8^+^ T cells in the PE was found between the resistant and sensitive groups (Figure 3B). Additionally, a significant difference was observed in Tim-3 expression in CD8^+^ T cells between the sensitive and control groups in the TME and PE (Figure 3B, C). However, no difference in Tim-3 expression in CD4^+^ T cells in the TME was found between the sensitive and control groups (Figure 3C).

We next performed LDH activity assays to assess the cytotoxicity of CTLs. The cytotoxic activity of CTLs in the sensitive group was significantly higher than that in the other groups (Figure 3D).

### Addition of anti-TIM-3 antibody overcame resistance to combination therapy with anti-PD-1 antibody and Anchored GM-CSF vaccine

**Figure 4:**
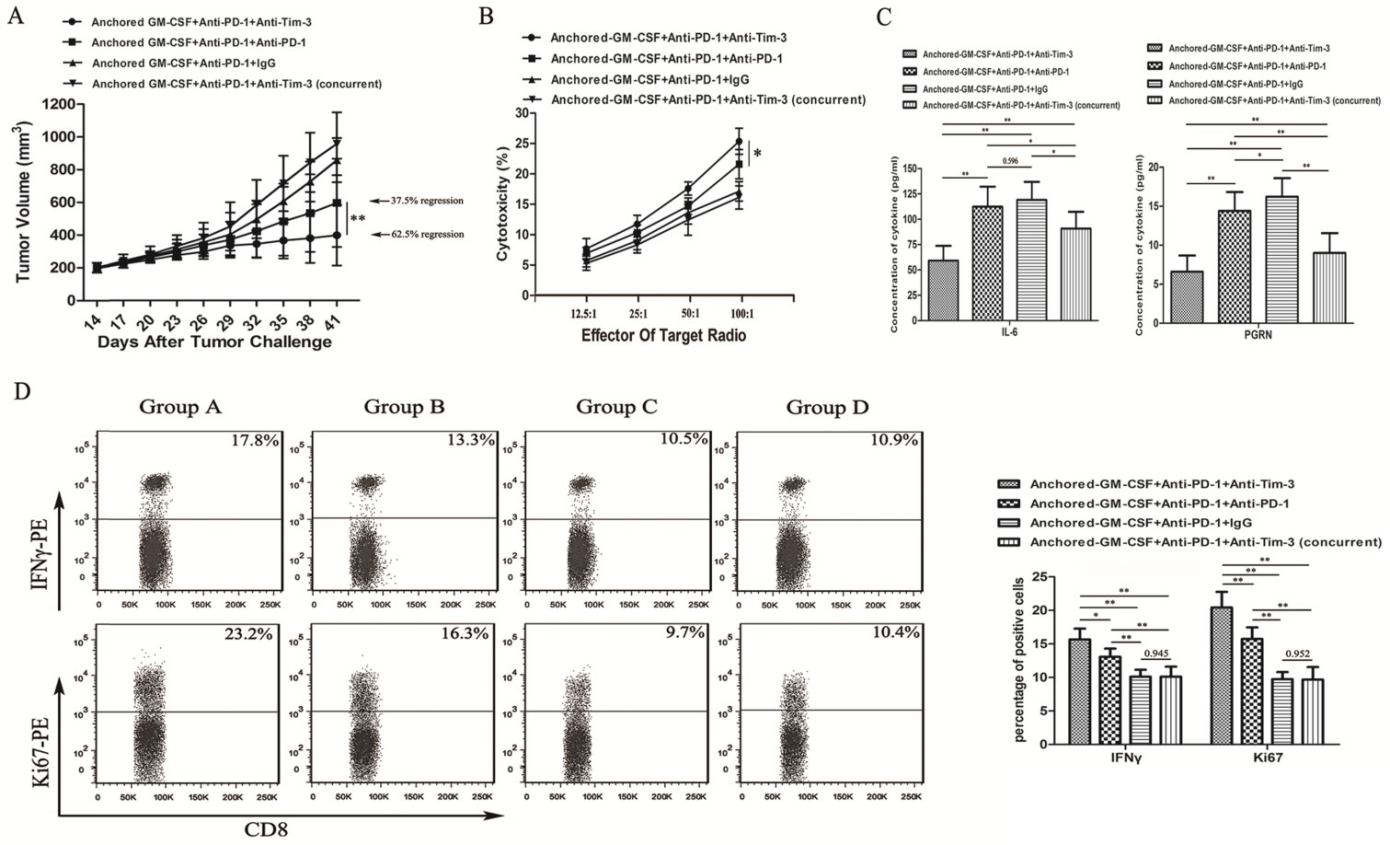
** Sequential administration of anti-PD-1 and anti-Tim-3 antibodies improved the efficacy of the Anchored GM-CSF vaccine therapy.** (A) All mice were subcutaneously injected with 2×10^5^ RM-1 cells, and the tumor volume was recorded. The sequential administration of anti-PD-1 and anti-Tim-3 antibodies with the Anchored GM-CSF vaccine further inhibited tumor growth compared with the other control treatments. The tumor regression rate in the experimental group increased to 62.5% (5/8). Concurrent PD-1 and Tim-3 blockade combined with the Anchored GM-CSF vaccine therapy did not show any significant advantage over the other treatments regarding antitumor effects. (B) After the treatment was finished, splenocytes were isolated from each group and stimulated with hIL-2. RM-1 cells served as target cells. (C) After the treatment was finished, peripheral blood was collected from each group and allowed to coagulate, and the supernatants were harvested by centrifugation. The concentrations of IL-6 (left) and PGRN (right) were measured by ELISA. (D) CD8^+^ T cells were isolated from tumor tissues from each group and purified by CD8 Microbeads. For proliferation analysis, CD8^+^ TILs were stained with anti-Ki67 antibodies and detected by flow cytometry (^*^P<0.05). For secretion function analysis, after stimulation with anti-CD3/CD28 beads and protein transport inhibitor for six hours, CD8^+^ TILs were stained with anti-IFNγ antibody and detected by flow cytometry. All experiments were repeated 3 times. ^*^P<0.05, ^**^P<0.01.

As we observed PD-1 blockade on tumor-infiltrating lymphocytes (TILs) and Tim-3 upregulation during resistance to the combination therapy with anti-PD-1 antibodies and the Anchored GM-CSF vaccine (day 26), we treated mice with an anti-Tim-3 antibody at this stage to investigate whether Tim-3 blockade can provide additional benefit against tumors (Figure 1B). As shown in Figure 4A, the sequential administration of anti-PD-1 and anti-Tim-3 antibodies combined with the Anchored GM-CSF vaccine further suppressed tumor growth and caused tumor regression in 62.5% of the treated mice.

To investigate the antitumor immunity, we determined the extent of cytotoxicity in each group using LDH activity in each well. Sequential administration of anti-PD-1 and anti-Tim-3 antibodies in combination with the Anchored GM-CSF vaccine treatment effectively improved the cytotoxic activity of CTLs (Figure 4B).

Some studies have found that checkpoint inhibition can affect immune-suppressive cytokine production in the TME 11. IL-6 has been recognized as a pleiotropic cytokine with an obvious tumor-promoting effect 17. PGRN is a regulator of tumorigenesis because it stimulates cell proliferation, migration, invasion, angiogenesis, malignant transformation, resistance to anticancer drugs, and immune evasion 18. In this study using the RM-1 tumor model, the concentrations of IL-6 and PGRN were significantly reduced with sequential treatment with anti-PD-1 and anti-Tim-3 antibodies (Figure 4C). To analyze proliferation and immune-promotive cytokine secretion, we isolated CD8^+^ TILs from each group and stained them with antibodies against Ki67 and IFNγ. The results of flow cytometry showed that the sequential administration of anti-PD-1 and anti-Tim-3 antibodies in combination with the Anchored GM-CSF vaccine significantly enhanced proliferation and IFNγ production in CD8^+^ TILs relative to those with other treatments (Figure 4D). Therefore, Tim-3 blockade might not only enhance the functions of antitumor CD8^+^ T cells but also decrease the levels of tumor-promoting cytokines.

## Discussion

The field of cancer immunotherapy has seen remarkable growth and renewed momentum in the past few years due to many major successes, most notably the development of T cell checkpoint inhibitors and therapeutic vaccines 3. PCa is a disease for which there has been little evidence of benefit following treatment with PD-1 blockade alone because of low tumor mutational burden 19 and rare PD-L1 expression on cancer cells 4. PD-L1 is an IFNγ-responsive gene, and PD-L1 expression in the TME is positively correlated with response to PD-1 blockade 20. Previously, we have shown that in RM-1 mouse models, the Anchored GM-CSF vaccine could effectively increase the frequency of tumor antigen-specific IFNγ-secreting CD8^+^ T cells, while PD-1 blockade could overcome the immune resistance associated with the Anchored GM-CSF vaccine treatment (modeling and treatment at the same time) 8. In this study, we simulated the clinical characteristics of treatment after tumor formation, which was different from our previous study. By observing the tumor size in each group, we found that combination therapy with anti-PD-1 antibodies and the Anchored GM-CSF vaccine could effectively reduce tumor growth and induce a better specific antitumor immune response than monotherapy with each agent. Notably, in this study, PD-1 blockade alone had no obvious therapeutic advantage over IgG treatment. This result was different from the finding in our previous study, and the possible reasons are that TILs are even more deficient in a tumor-forming environment and that preexisting antitumor T cells in the TME are necessary for the efficacy of PD-1 blockade 21.

In this study, although combination therapy with anti-PD-1 antibodies and the Anchored GM-CSF vaccine induced a robust antitumor immune response, some of the mice still exhibited eventual tumor progression, and tumor regression occurred in only a few mice. This finding was consistent with those of others, suggesting that “checkpoint” blockade alone cannot completely reverse immune resistance because of activation of compensatory pathways 11,22. Some studies have shown that Tim-3 is co-expressed with PD-1 in exhausted T cells in the context of antimicrobial responses 23,24. In addition, in mice lacking systemic PD-1 in the context of acute myeloid leukemia (AML), T cells express high levels of Tim-3 12, supporting the notion that compensatory pathways are upregulated in the absence of PD-1. In this study, to investigate Tim-3 expression on T cells after PD-1 blockade, we divided the mice (in the Anchored GM-CSF vaccine+anti-PD-1 antibody group) into sensitive and resistant groups according to the change in tumor size. We found that Tim-3 expression was significantly higher on TILs from the resistant group than on TILs from the sensitive and control groups. This compensatory upregulation of Tim-3 expression in the TME may be a specific adaptive response to sustain the dysfunction of TILs.

Our study showed that PD-1 blockade could induce the upregulation of Tim-3 expression in TILs, and the expression of Tim-3 could decrease the antitumor activity of T cells. We subsequently combined the Anchored GM-CSF vaccine with PD-1 and Tim-3 blockade in an established subcutaneous PCa model and found that this combination therapy could significantly suppress tumor growth and increase the tumor regression rate in 62.5% of the mice. The improvement in the proliferation, secretion, and cytotoxic activity of CD8^+^ TILs and a reduction in tumor-promoting cytokine production indicated that the sequential administration of anti-PD-1 and anti-Tim-3 antibodies with the Anchored GM-CSF vaccine induced a highly robust antitumor immune response. Unlike other models where concurrent blockade of PD-1 and Tim-3 delayed tumor progression as initial therapy in the context of high expression levels of these checkpoint proteins 25, we observed no additional benefit of the combination therapy (simultaneous Tim-3 and PD-1 blockade) as initial therapy over PD-1 blockade alone. This finding was consistent with the study by Shohei Koyama, suggesting that concurrent PD-1 and Tim-3 blockade did not show any significant advantage regarding antitumor effects in mouse models of lung adenocarcinoma 11. The reason for this may be that Tim-3 was not acutely upregulated at the time points when we confirmed clinical efficacy 26, and Tim-3 positivity was significantly correlated with the duration of PD-1 blockade 11. In the combined treatment group (sequential administration of PD-1 and Tim-3 blockade), tumor growth could not be effectively inhibited in some mice. This result suggested that additional immune checkpoints (CTLA-4 or LAG-3) may be upregulated in response to combination therapy with anti-PD-1 and anti-Tim-3 antibodies, which might also limit therapeutic activity.

Our study had some limitations that should be considered when interpreting our results. First, we focused on overcoming vaccine-induced T cell exhaustion/dysfunction in a PCa model (RM-1 model). T cell exhaustion/dysfunction is a double-edged sword and promoted mainly by tumors. Therefore, the most accurate approach is to verify the results in multiple tumor cell lines. Second, in this study, we followed the principles of Response Evaluation Criteria in Solid Tumors (RECIST) for human solid tumors. Resistance in our study was defined as an initial therapeutic response of tumors followed by growth to >120% of the original tumor size. This was only a rough definition and assessment. The more accurate definition of response/resistance in mouse studies requires further refinement and investigation. Third, in our study, Tim-3 expression on TILs was significantly upregulated in the group resistant to PD-1 blockade. However, the specific relationship between Tim-3 expression on TILs and PD-1 blockade and the best time point of treatment via Tim-3 blockade in PCa (RM-1 model) need to be further studied. Fourth, this is a preclinical study, and we will use human PCa cells for more in-depth research and investigation in the future.

In summary, in this study, we demonstrate that the decrease in the therapeutic effect of the Anchored GM-CSF vaccine combined with anti-PD-1 antibodies is related to the upregulation of Tim-3 expression on TILs and that Tim-3 blockade following the Anchored GM-CSF vaccine and anti-PD-1 antibody therapy can further overcome adaptive immune resistance in established PCa.
